# Proteomics dataset for the analysis of the effects of Grammatophyllum speciosum extracts on RAW 264.7 cells

**DOI:** 10.1016/j.dib.2023.108937

**Published:** 2023-01-26

**Authors:** Yodying Yingchutrakul, Sucheewin Krobthong

**Affiliations:** a National Omics Center, NSTDA, Pathum Thani 12120, Thailand; b Center of Excellence in Natural Products Chemistry (CENP), Department of Chemistry, Faculty of Science, Chulalongkorn University, Bangkok 10330, Thailand

**Keywords:** Tiger orchid, Macrophage, LC-MS/MS, Traditional plant

## Abstract

Grammatophyllum speciosum is a traditional plant with beneficial functionalities for health. G. speciosum extracts can inhibit collagenase and nitric oxide without cellular toxicity in keratinocytes. The extracts have shown potential for use and formulation as cosmeceutical ingredients. However, the molecular mechanisms underlying these activities remain unknown. In this dataset, we used a proteomics approach to clarify the proteins that participate in the response of RAW264.7 macrophage cells to G. speciosum extracts. Cells were divided into two experimental groups, i.e., the control and treatment groups. In turn, the treatment group included two subgroups that were treated with 20 and 100  µg/mL of the extracts, respectively. The experiments were conducted using two biological replicates. The dataset was obtained from label-free proteomics using high-resolution tandem mass spectroscopy (LC-MS/MS) with four technical replicates. The quality control (QC) of the proteomics dataset was carried out using chromatography at the MS1 and MS2 levels, peptide mass deviation, peptide mass cleavage, sequence length, and total peptide intensity. The global proteome profile was analyzed using a principal component analysis (PCA). These datasets can clarify the potential pathways or proteins involved in the response to the extracts, to support their potential applicability for the development of cosmeceutical ingredients.

**Specifications Table**

**Table:** 

Subject	*Omics: Proteomics*
Specific subject area	*Proteomics*
Type of data	Figure RAW data Supplementary Table (excel)
How the data were acquired	*Q Exactive HF Hybrid Quadrupole-Orbitrap inline coupled to nano-LC Dionex Ultimate 3000RSLC high-performance liquid chromatography system (Thermo Scientific) was used to acquie the data using data dependent method (DDA).*
Data format	Raw data from LC-MS/MS Analyzed files
Description of data collection	The cells lysate preparation by denaturation, reduction, and alkylation of the proteins (2-biological replication).The denatured proteins are digested into tryptic peptides. Next, the tryptic peptides were fractionated using C18-reverse phase chromatography and subjected to mass spectroscopy (4-injection/replication)*.* Tryptic peptide ions were identified from the mass spectra through sequence database searching. To quantify the proteins levels across the treatment (20 and 100 µg/mL of extract) and control conditions, we used label-free quantification.
Data source location	Institution: Mahidol University City: Bangkok Country: Thailand
Data accessibility	Mass spectrometry data have been deposited to the ProteomeXchange Consortium via the PRIDE repository Data identification number: PXD039206 Data DOI: 10.6019/PXD039206 URL: http://www.ebi.ac.uk/pride/archive/projects/PXD039206
Related research article	*Yingchutrakul Y, Sittisaree W, Mahatnirunkul T, Chomtong T, Tulyananda T, Krobthong S. Cosmeceutical Potentials of Grammatophyllum speciosum Extracts: Anti-Inflammations and Anti-Collagenase Activities with Phytochemical Profile Analysis Using an Untargeted Metabolomics Approach. Cosmetics. 2021; 8(4):116*. https://doi.org/10.3390/cosmetics8040116

## Value of the Data

• High-resolution mass spectroscopy-based proteomics data are important because they represent the first comprehensive dataset on the response of macrophage cells to G. speciosum extracts.
• Both the public and private sectors can benefit from these data, as the use of anti-inflammatory products extracted from G. speciosum requires substantial investment and infrastructure.
• These data can be used by researchers to determine whether a specific protein biomarker exists in the data set. The data can be used to identify potential pathways in support and for the improvement of a cure/diagnosis, as well as potential targets for cosmeceutical ingredient development.
• This dataset enables the independent assessment of the remaining molecular aspects of the results described in scientific publications.

## 1 Objective

The identification of novel natural products for use in cosmetics remains a challenge. The generation of molecular information is important as the first step of product development. The dataset can function as the fundamental knowledge for the development of natural anti-inflammatory ingredients from G. speciosum extracts that can promote skin recovery and the healing of blemished skin.

## 2 Data Description

This article presents a dataset generated from the comparison of the proteome of RAW264.7 cells in response to G. *speciosum* extracts. The instrument and sample processing were qualified, to ensure the reliability of the dataset. Therefore, we analyzed the MS1 and MS2 spectra. The integrated peak intensities of precursor ions (MS1) for different experimental conditions were obtained from separate LC-MS runs and were used to compare the relative protein expression levels between experimental groups. In some cases, the LC-MS runs led to variations, including inconsistencies between the chromatography peak and the total number of ions (TICs). Therefore, the consistency of the MS1 and MS2 chromatograms were assessed (Table 1).

**Table 1:** TIC data based on the MS1 and M2 intensities of all LC-runs.

Experimental group	Biological replicates	Technical replicates	MS1 TIC intensity (×10^9^ AU)	MS2 TIC intensity
Control	1	1	0.908	1.17
		2	1.25	1.83
		3	1.42	2.04
		4	1.53	1.27

	2	1	1.22	1.42
		2	0.946	1.02
		3	0.901	1.65
4		1.21	1.35

Treatment: 20 µg/mL	1	1	1.17	1.79
		2	1.20	1.99
		3	0.801	1.81
		4	1.31	2.06

	2	1	0.997	1.62
		2	1.26	2.07
		3	1.01	1.90
4		0.956	2.13

Treatment: 100 µg/mL	1	1	1.35	1.48
		2	1.11	1.83
		3	1.52	1.98
		4	0.966	1.69

	2	1	0.940	1.67
		2	0.961	2.31
		3	1.13	1.98
4		1.01	2.18

**Fig. 1:**
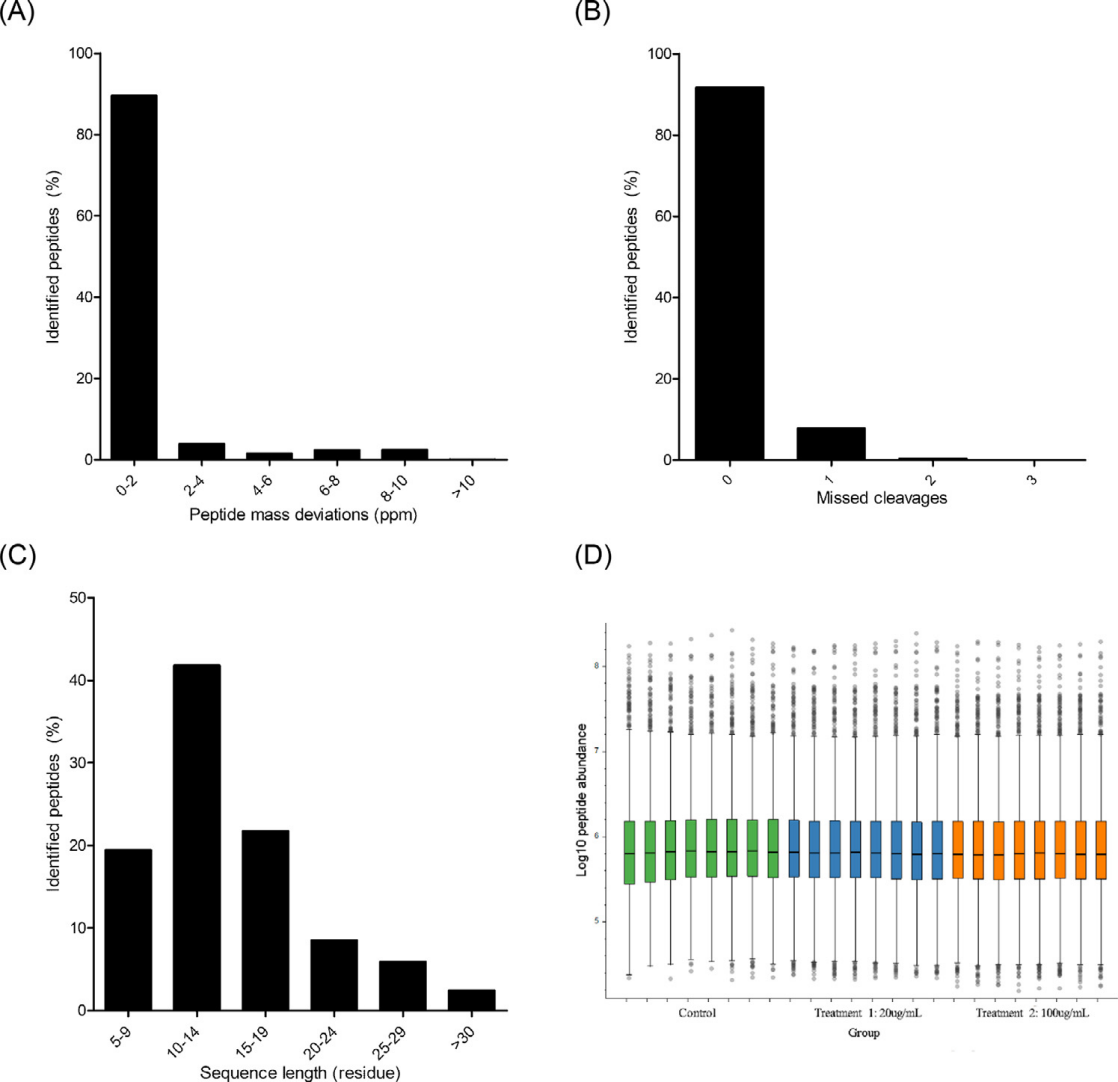
QC of proteome data. (A) Deviation of the mean tryptic peptide m/z. (B) Percentage of peptide cleavage sites. (C) Percentage of tryptic peptide size distribution. (D) Box-plot analysis showing the distribution of the sum of the identified peptide intensities in each experimental sample (the green, blue, and orange boxes represent the control, treatment 1, and treatment 2, respectively). The plot includes the upper (Q1) and lower (Q3) quartiles) with the median value indicated by the horizontal line.

The overall MS1 and MS2 intensities exhibited a small variation (standard deviation, 0.202 and 0.337 for MS1 and MS2, respectively) between the two independent experiments and the four technical replicates. This result suggests that the total intensities of the 24 LC-MS runs had a high consistency. The consistent correlation between MS1 and MS2 may reflect the consistent reproducibility of the sampling process and the MS setting parameters. The proteins identified in the experiments are listed in Supplementary Table S1. In addition, Supplementary Table S2 lists the peptides that were identified from each protein. To increase confidence in the results obtained, proper quality control (QC) measures are necessary to monitor and control the existing variation. Peptide mass deviations are important parameters related to mass accuracy in proteomics data. As shown in Fig. 1A, our results showed that >89.0% of the identified peptides had a low mass deviation (<2 ppm).

The deviation in peptide mass was strictly set at 0–10 ppm. The small-mass deviation cutoff value of a peptide acts as a filter and directly reduces the number of false-positive peptides. Moreover, the effectiveness of the proteolytic digestion stability of trypsin was studied. As depicted in Fig. 1B, our results revealed that 91.7% of the identified peptides also did not exhibit missed cleavage peptides. The length of the identified peptides was within 8–20 residues (Fig. 1C). Regarding the quantification aspects, label-free proteomics is being used increasingly to estimate relative protein abundances in different experimental conditions [1]. However, the label-free technique strictly requires normalization to improve the accuracy of the measurement of total proteins. Another QC consisted of the estimation of the intensities of all identified peptides, as measured from a proteome list (Supplementary Table S1). Our results identified 6452 peptide groups (as shown in Supplementary Table S2), whereas the sum of the intensities of the identified peptides is reported in Fig. 1D.

Trypsin is a general and well-known protease that is used in proteomics experiments [2]. The molecular mass of the tryptic peptides was within the optimum mass range and was suitable for LC-MS/MS analysis (based on an in-silico digestion of all proteins in the UniProtKB database) [3]. The in-silico experiment reported that full-length tryptic peptides comprised a median value of 12 residues (inter-quartile range, 8–20 residues) [4]. Therefore, the level of mass deviation, missed cleavage peptide, tryptic peptide length, and total peptide abundance were qualified. These QC analyses suggested that our proteomics dataset is generally applicable for differential protein expression analysis.

**Fig. 2:**
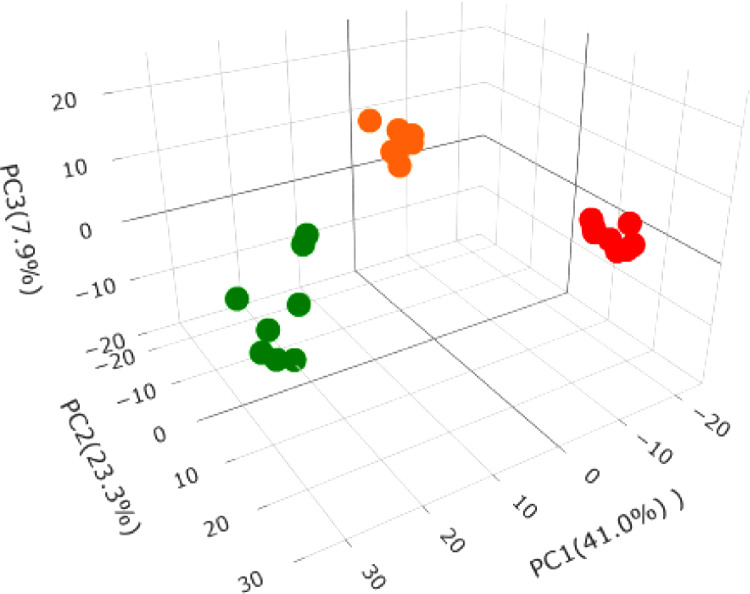
PCA of proteome data. Different treatment conditions are indicated by different colored circles. The control is indicated as green circles, treatment with extract at 20 μg/mL is shown as orange circles and treatment with extract at 100 μg/mL is shown as red circles.

Proteomics is a very sensitive method for identifying and quantifying proteins. Relatively abundant proteins with false discovery rate (FDR) values <0.01 among the 24 LC-MS runs were considered as being differentially expressed. A total of 1712 proteins were successfully identified. To explore the variance in the proteome profiles, a PCA analysis was used to compare the three experimental groups of 24 LC-MS runs, to assess the intra- and inter-experimental variations. A PCA of the two independent biological and four technical replicates of each group was performed, as shown in Fig. 2.

The treatment groups (20 and 100 µg/mL subgroups) were clustered in different regions compared with the control group. Groups of close-group samples showed a strong correlation between the protein expression patterns of each condition, whereas distant samples exhibited a weaker correlation. The PCA analysis revealed that the treatment and control groups were completely separated, indicating differences in their protein profiles.

## 3 Experimental Design, Materials and Methods

*The equipment used in this study was as follows:* UltiMate™ 3000 UHPLC Systems, Q Exactive HF mass spectrometer, temperature-controlled incubator (heat block), temperature-controlled bench-top centrifuge, and SpeedVac Concentrator, which were purchased from Thermo Scientific Co. (Waltham, MA, USA). The materials used for sample preparation and cleaning-up were as follows: low protein binding microcentrifuge tubes, Pierce Detergent Removal Spin Columns (0.5 mL), trapping column, RSLC C18 (0.5 cm × 75 um), and EASY-SPRAY PEPMAP RSLC C18 2 um, (25 cm × 75 um, 2 um), which were purchased from Thermo Scientific Co. The LC-MS vial (TruView) was purchased from Waters Co. (Milford, MA, USA). The chemicals and solvents used here were as follows: cell lysis buffer (0.5% SDS, 5 mM TCEP, 10 mM NaCl in 40 mM HEPES-KOH pH 8.0, with 1× protease inhibitor cocktail; freshly prepared), trypsin-compatible solubilization buffer (0.2% RapiGest SF, 5 mM NaCl in 10 mM NH_4_HCO_3_; freshly prepared), reduction buffer (10 mM TCEP in 10 mM NH_4_HCO_3_), alkylation buffer (25 mM Iodoacetamide in 10 mM NH_4_HCO_3_; freshly prepared in an amber tube). The solvent used for LC-MS/MS: Water with 0.1% (v/v) formic acid and acetonitrile with 0.1% (v/v) formic acid, which was purchased from Merck Co., all chemicals and buffers were prepared using LC-MS-grade water purchased from Merck Co.

### 3.1 Protein extraction, cleaning-up, and preparation for proteomics analysis

The effect of the *G. speciosum* extract on RAW264.7 cells was investigated. The experiment was conducted using a previously published protocol without any modification *[**5**]*. Briefly, the cells were treated with (20 and 100 µg/mL) of the extracts and culture medium as control for 24 h. The cell pallet was collected and washed using a 1× PBS solution, with three replicates (including two biological replicates per condition). Lysis buffer was added and the cell pellet was resuspended cell pellet on ice, followed by sonication using a probe tip in 250  µL of cell lysis buffer at a frequency of 20 kHz and 80% amplitude for 2 s “on” and 3 s “off” for a total of 20 s. The protein lysate was incubated at room temperature for 10 min, then transferred into 1.5 mL centrifugation tubes and centrifuged at 15,000 × *g* for 20 min at room temperature. The supernatant was collected and aliquoted for protein determination using the BCA protein assay. The protein concentration of all samples was adjusted to 1 µg/µL. A total of 20 µg of protein in solution (20 µL) was used for reduction and alkylation. Subsequently, 5 µL of the reduction solution was added (to obtain a final concentration of TCEP of 2 mM) and the solution was incubated at 45°C for 60 min, followed by cooling down, the addition of 25 µL of alkylation solution (to obtain a final concentration of IAA of 12.5 mM), and incubation at room temperature (in the dark) for 40 min. The sample was cleaned-up using Pierce Detergent Removal Spin Columns (0.5 mL) and the flow-through fraction was collected. Next, 5 µL of RapiGest solution (1% RapidGest SF in 10 mM ammonium bicarbonate) was added and incubated for 5 min at room temperature. Proteolytic digestion was conducted by adding 5 µL of a 40 ng/µL Trypsin/LysC solution (in 10 mM ammonium bicarbonate) and incubating at 37°C for 4 h. The digestion reaction was stopped by adding 6 µL of 1% formic acid. The tryptic peptides were dried using a speed vacuum and resolved in 80 µL of water in 0.1% formic acid by pipetting up and down 20 times, followed by 30 s of vortexing, to ensure full solubilization of the peptides. The solution was centrifuged at 15,000 × *g* for 30 min at room temperature. A total of 20 µL of the solution was transferred to an LC-MS vial.

### 3.2 LC-MS/MS configuration

A mass spectrometer combined with an UltiMate 3000 LC system was used. A total of 1.01 µg of the tryptic peptide was loaded onto the trapping column and the analysis was performed at a flow rate of 10 µl/min for 1.5 min before the column was switched in line with the analytical column (C18 PepMapTM 100 capillary column). The tryptic digest was resolved onto the analytical column with 120 min gradients, as described below (Table 2).

**Table 2:** LC gradient program used for the elution of the K562 cell digest during the LC-MS/MS analysis.

Time (min)	Flow rate (nL/min)	%MP A^a^	%MP B^b^
Initial 2.00 100 101 125 145 150 151 185 (Stop run)	300 300 300 300 300 300 300 300 300	98 98 75 75 55 15 15 98 98	2 2 25 25 45 85 85 2 2

a Mobile phase A consisted of 0.1% formic acid in water.

b Mobile phase B consisted of 0.1% formic acid in acetonitrile.

For this LC- setup, sample loading onto the column was performed using a 20 µL loop and for all analyses, 4 µL were directly injected into the LC system using the microliter pick-up method. Between samples, a blank (0.1%formic acid in water) was run to prevent sample carry-over. Protonated tryptic peptides were formed in electrospray ionization (ESI) and transferred to mass spectroscopy during the nebulization process using 1.9 kV at 300°C at the end of the nebulizer needle tip. The normalized collision energy was 30 for higher collisional dissociation. The MS data were acquired in a data-dependent mode, as described below (Table 3).

**Table 3:** Mass spectroscopy settings used in the proteomics analysis.

MS method parameter	Value
1. MS event 1.1 Full MS Settings Maximum injection time (IT) Resolution Automatic gain control (AGC) target Scan range *m/z* 2. MS2 event 2.1 Fragmentation Settings Number of precursors Normalized collision energy Dynamic exclusion Precursor exclusion Exclusion threshold Isolation window width Charge state 2.1 MS2 Settings Max IT Resolution AGC target	30 ms 120 k 3e^6^ 400–1,500 15 27 True 25 10 1.4 2–5 50 ms 15 k 10 k

### 3.3 Protein identification and quantification

The raw mass spectra (.raw file) were processed using Proteome Discoverer 2.4. The setting parameters for protein identification were as follows: parent ion mass error tolerance, 10 ppm; fragment ion mass error tolerance, 0.05 Da; minimum fragment ion matches per peptide, 3; digesting enzyme, trypsin (Full); one fixed modification: carboxyamidomethylation of Cys; and two variable modifications: deamidation of Gln and Asn and oxidation of Met. The peptide spectrum was matched to the *Mus musculus* reviewed database (17,144 sequences, UniprotKB, accession date: 2 January 2022). The FDR of the peptides was set to 1%. Digestion specificity was measured by investigating peptides with missed cleavage sites and the length of the tryptic digestion products. The data that passed the QC were subjected to downstream analysis. The total ion count in each injection was analyzed. The normalization of the relative protein abundance ratio was performed using the total peptide amount for each LC run (across all runs; n = 48) via a normalization algorithm (total intensity count) of the PD software. To assemble a differentially expressed protein list, multiple consensus workflows were used within the PD to compile the peptide spectrum matches into peptide groups, protein database matches, and non-redundant protein groups using the principle of strict parsimony, as defined by the software default settings. To investigate the heterogeneity of the variation of the experimental conditions of cells, a PCA was used to visualize the intra- and inter-group replicate differences. The proteomics data were deposited in the ProteomeXchange Consortium via the PRIDE partner repository [6].

## Ethics Statement

This study does not involve experiments on humans or animals.

## CRediT authorship contribution statement

**Yodying Yingchutrakul:** Conceptualization, Methodology, Investigation, Writing – original draft, Writing – review & editing. **Sucheewin Krobthong:** Conceptualization, Writing – review & editing, Supervision, Project administration, Funding acquisition.

## Declaration of Competing Interest

The authors declare that they have no known competing financial interests or personal relationships that could have appeared to influence the work reported in this paper.

## Data Availability

Proteomics analysis of the effects of Grammatophyllum speciosum extracts on macrophage cells (Original data) (ProteomeXchange Consortium via the PRIDE).

## Appendix Supplementary materials

Image, application 1

Image, application 2
